# Application of the principles of evidence-based practice in decision making among senior management in Nova Scotia’s addiction services agencies

**DOI:** 10.1186/1747-597X-9-47

**Published:** 2014-12-05

**Authors:** Matthew Murphy, M Jayne MacCarthy, Lynda McAllister, Robert Gilbert

**Affiliations:** Department of Quality Management, Guysborough Antigonish Strait Health Authority, 25 Bay Street, Antigonish, NS B2G2G5 Canada; Quality and Research Utilization Team, Addiction Services, Pictou County Health Authority, 199 Elliott Street, Pictou, NS B0K1H0 Canada; School of Health Sciences, Dalhousie University, 1278 South Park Street, Halifax, NS B3H 2Y9 Canada

**Keywords:** Evidence-based decision-making, Evidence-based medicine, Substance abuse, Addiction, Treatment, Prevention, Senior Administrative Personnel, Qualitative research

## Abstract

**Background:**

Competency profiles for occupational clusters within Canada’s substance abuse workforce (SAW) define the need for skill and knowledge in evidence-based practice (EBP) across all its members. Members of the Senior Management occupational cluster hold ultimate responsibility for decisions made within addiction services agencies and therefore must possess the highest level of proficiency in EBP. The objective of this study was to assess the knowledge of the principles of EBP, and use of the components of the evidence-based decision making (EBDM) process in members of this occupational cluster from selected addiction services agencies in Nova Scotia.

**Methods:**

A convenience sampling method was used to recruit participants from addiction services agencies. Semi-structured qualitative interviews were conducted with eighteen Senior Management. The interviews were audio-recorded, transcribed verbatim and checked by the participants. Interview transcripts were coded and analyzed for themes using content analysis and assisted by qualitative data analysis software (NVivo 9.0).

**Results:**

Data analysis revealed four main themes: 1) Senior Management believe that addictions services agencies are evidence-based; 2) Consensus-based decision making is the norm; 3) Senior Management understand the principles of EBP and; 4) Senior Management do not themselves use all components of the EBDM process when making decisions, oftentimes delegating components of this process to decision support staff.

**Conclusions:**

Senior Management possess an understanding of the principles of EBP, however, when making decisions they often delegate components of the EBDM process to decision support staff. Decision support staff are not defined as an occupational cluster in Canada’s SAW and have not been ascribed a competency profile. As such, there is no guarantee that this group possesses competency in EBDM. There is a need to advocate for the development of a defined occupational cluster and associated competency profile for this critical group.

**Electronic supplementary material:**

The online version of this article (doi:10.1186/1747-597X-9-47) contains supplementary material, which is available to authorized users.

## Background

### Canada’s substance abuse workforce: present and future

Canada’s substance abuse workforce (SAW) is an unregulated profession. At the national level its development is supported by the Canadian Centre on Substance Abuse (CCSA) which provides evidence-based analysis and advice to mobilize collaborative efforts to reduce alcohol and other drug-related harms.

CCSA conducted a nationwide study of 2,720 front line staff in 2004 [1], and determined that there was no consistent level of expertise among substance abuse professionals in Canada. CCSA conducted a follow up study in 2010, under the guidance of the National Advisory Group on Workforce Development, and determined that most addictions prevention and treatment agencies in Canada are staffed by persons working within one of seven occupational clusters (job groups). Identification and characterization of these occupational clusters were developed through research and consultation with subject matter experts and validated through focus groups (of professionals working within these occupational clusters) from all Canadian provinces and territories. The seven clusters and their defined roles and responsibilities are presented in the document *Competencies for Canada’s Substance Abuse Workforce*[2] and include: Administrative Support; Counsellor; Health Promotion; Senior Management; Supervision; Support and Outreach and Withdrawal Management.

### Competency profiles and Canada’s substance abuse workforce

In Canada, agencies that prevent and treat substance related and addiction disorders deliver care through *systems*[3] supported by evidence-informed policies, guidelines, standards, programs and services. By their nature, the development, implementation and sustainability of effective *systems* require constructive and evidence-informed contributions from all members of all “occupational clusters” within the *system*. For this to occur, all contributors must possess competency in the substance related prevention and treatment field. Competency is defined as “specific, measurable skills, knowledge, attitudes, and values needed to effectively perform a particular job function or role. They are typically learned and developed through work, education, training, and other life experiences” [2] (pI-4). CCSA defines core competency as: common required skills, knowledge and attitudes across all those working in the substance related and addiction disorder field. Specialty competency is defined as: knowledge, skills and attitudes required to judiciously fulfill practice roles within one’s own occupational cluster.

The 2005 study entitled *Optimizing Canada’s Substance Abuse Workforce: Results of a National Survey of Service Providers*[2] determined that there was no consistent level of expertise among Canada’s SAW. This work demonstrated a crucial need for the identification and development of competencies, both *core* and *specialty*, required to perform and contribute effectively within addiction *systems*.

As an initial response to these defined needs, CCSA, under the guidance of the National Advisory Group on Workforce Development, set out to identify *core* competencies for Canada’s SAW. Through research and consultation with subject matter experts across Canada, two sets of *core* competencies: technical and behavioral were developed. CCSA defines technical competencies as “the knowledge and abilities required when applying specific technical principles and information in a job function or role” [2](pii). Behavioral competencies are defined as “abilities, attitudes and values required to perform effectively in a job function or role” [2](pii).

Subsequent consultation, with approximately 120 people in focus groups across Canada, lead to the validation of the behavioural competencies, and identified appropriate proficiency levels for the seven occupational clusters described for Canada’s SAW. The work was presented in the 2010 document *Competencies for Canada’s Substance Abuse Workforce*[2]; the results have been summarized in Table 1. (used with permission, see Additional file 1).

**Table 1:** **Behavioral competency profiles and levels of proficiency by occupational cluster (used with permission see Additional file** **1** **)**

	Levels of proficiency by occupational cluster						
Competencies	Administration support	Counseling	Health promotion	Senior management	Supervision	Support & outreach	Withdrawal management
Adaptability/Flexibility	1	3*	3	4**	3*	3	3
Analytical Thinking and Decision making	1*	3**	2*	4*	3*	2*	2*
Client-centered Change		3*				3	3*
Client Service Orientation	1*	2**			3*		
Collaboration and Network Building			3*	4*		2*	
Continous Learning	2	2	3	4	3	2	2
Creativity and Innovation			3	4*	3*	2**	
Developing Others			3*	4**	3		
Diversity and Cultural Responsiveness	1	3	3	4	3	3	3
Effective Communication	2	3	3	4	3*	3	3
Ethical Conduct and Professionalism	1	2	2	4	3	2	2
Interpersonal Rapport/Savvy	2	3*	3*		3**	3*	3*
Leadership				4	3*		
Planning and Organizing	2		3*	4*	3*	2	3*
Self Care	2	3	2	4*	3	3	2
Self Management	2	2**				2*	3*
Self Motivation and Drive			2**				3*
Teamwork and Cooperation	2	2*	2*	4**	3**		2

Behavioral Competency Profiles.

1 = Introductory level, 2 = Basic level, 3 = Intermediate level, 4 = Advanced level.

*indicates 80% agreement, **indicates 60% agreement, Blank cell indicates less than 60% agreement.

Number on its own indicates 100% agreement.

Four levels of proficiency (introductory, basic, intermediate and advanced) have been developed for the behavioural competencies. These are intended to define the competencies and the degrees of proficiency in those competencies for persons working within specific occupational clusters. To support the definition of degree of proficiency, lists of behavioural *indicators* have also been identified. These indicators are examples of successful (observable) performance in the competencies.

According to CCSA [2], the development of competencies in members of Canada’s SAW will enhance professionalism and excellence within the substance-related and addictive disorder field by defining the required knowledge, values and skill sets. Furthermore, defined competencies will support the development of evidence-based prevention and treatment *systems* and assist employers in the hiring and developing of staff (i.e. design of education and training curriculum that is responsive to expectation and need). Ultimately this will provide Canadians with a more consistent quality of service from its substance and addiction prevention and treatment agencies.

### Aptitude in evidence-based practice is essential to the attainment of behavioural competencies

Although behavioural competencies are not explicitly taught, there are certain technical competencies that a person must possess in order to be proficient in the ascribed behavioural *indicators*. Included among these is competency in Evidence-Based Practice (EBP). Evidence-Based Practice is defined, in the context of this paper, as “a formalized process of comprehensively using the skills for identifying, searching for and interpreting the results of best scientific evidence, considered in conjunction with relevant expertise (experience and judgment), the client’s preference’s and values, and the context within which decisions are being made”. A review of the indicators for the behavioural competencies for Canada’s SAW reveals that both knowledge of the principles of EBP, as well as aptitude in the skills required to apply these principles is essential to achieve a basic level proficiency in twelve of the eighteen behavioural competencies. Descriptions of these competencies are provided in Table 2[2] and demonstrate that all occupational clusters possess, within their profile, a requirement for competency in EBP. Furthermore, persons classified as Senior Management, being ultimately responsible for all decisions pertaining to policies, standards, programs, practice guidelines and services, require high levels of proficiency in many competencies requiring knowledge of, and skill in, EBP. Indicators associated with the competencies requiring knowledge of, and skill in EBP for Senior Management, are provided in Additional file 2. To date, no comprehensive study has evaluated the knowledge of the principles of EBP or the use of the components of the evidence-based decision making (EBDM) process in Canada’s SAW.

**Table 2:** **Behavioural** ***competencies requiring knowledge and use of EBP*** **(Used with permission** [4] **)**

Competency title	Competency description
Adaptability/Flexibility	Willingly adjust one’s approach to meet the demands of constantly changing conditions, situations and people and to work effectively in difficult or ambiguous situations
Analytical Thinking and Decision Making	Gather, synthesize and evaluate information to determine possible alternatives and outcomes and make well-informed, timely decisions. Includes critical thinking and reasoning skills.
Continuous Learning	Identify and pursue learning opportunities to enhance one’s professional performance and development and the effective delivery of high-quality programs and services.
Creativity and Innovation	Using evidence-based practices in innovative and creative ways to initiate both effective new ways of working and advances in the understanding of the field of practice. Innovation and creativity are achieved in translating research into practice to optimize improvements in service delivery and professional practice.
Developing Others	Facilitate and motivate sustained learning and create opportunities and resources, as well as promote and respect others’ needs for ownership of learning outcomes. Includes creation of a continuous learning environment that fosters positive growth in both work and public contexts among peers, clients, client families, communities, and other groups (recipients).
Effective Communication	Articulates both verbally and in writing across a range of technologies in a manner that builds trust, respect and credibility and that ensures the message is received and understood by the audience. Includes active listening skills and congruent non-verbal communication.
Self Care	Deliberately and continuously apply professional and personal self care principles to oneself and, at times, others to sustain optimal productivity while maintaining physical, mental, spiritual and emotional health.
Leadership	Help others achieve excellent results and create enthusiasm for a shared vision and mission, even in the face of critical debate
Ethical Conduct and Professionalism	Provide professional services according to the principles and values of integrity, competence, responsibility, respect, and trust to safeguard both self and others. Includes the development of professionalism and ethical behavior in self and others (individuals, groups, organizations, communities).
Self Motivation and Drive	Remain motivated and focused on a goal until the best possible results are achieved, with both passion for making a difference in the substance abuse field and persistence despite confronting obstacles, resistance and setbacks.
Client-Centred Change	Enhance, facilitate, support, empower and otherwise increase client motivation for positive change. Positive change is achieved by involving the client actively in the change process and encouraging the client to take responsibility for outcomes he or she achieves. Clients may be individuals, groups, communities and organizations.
Client Service Orientation	Provide service excellence to clients (which can include individuals, groups, communities and organizations). Includes making a commitment to serve clients focusing one’s efforts on discovering and meeting client’s needs within personal, professional and organizational capacities and boundaries.

### Evidence-based practice

#### Historical review

Prior to the 1970’s health-care related decision-making did not exist as a field of study [5]. In 1973 a paper was published [6] documenting wide variations in practice among physicians; the authors discovered that when different physicians were faced with essentially similar patients, they did not in fact, make similar recommendations. Subsequent to these findings, researchers at the Research and Development Corporation published a series of studies in the 1980’s highlighting the fact that a significant number of procedures performed by physicians were inappropriate, even when considered by the standard of physician experts [7]. Another major concern noted was the significant lag time between clinical research and its application to clinical practice. It has been estimated that during this time period only 15 percent of medical practices were based on clinical trials [7]. Further, as the use of clinical trials increased, it was discovered that many of the procedures being performed by physicians were ineffective.

The above mentioned factors helped to identify a need that would eventually be addressed through the work of the Evidence-Based Medicine Working Group, led by Gordon Guyatt [8]. Beginning in the late 1980s this group would develop the philosophy of modern day EBP and its application process EBDM. Its success in medicine lead to its integration into the competency profiles of most other health care professions, and its principles are now reflected in the curriculum framework of all Canadian degree-based health programs. In addition to helping the practitioner, the EBDM process has also provided a valuable tool in its capacity to support, at the organizational level, many aspects of healthcare decision-making. In today’s field of health care, the EBDM process is frequently employed in the development of programs, policies, clinical practice guidelines, and standards and best practices. In recognition of its importance to many areas of healthcare, the definition of EBM has been broadened and the term EBP is now routinely employed.

Today the EBDM process helps decision-makers in health care to collect and critically appraise all best evidence for the purpose of guiding decision making. A number of benefits have been ascribed to the use of EBP. These include, but are not limited to: increases in treatment quality, effectiveness and consistency in practice; better use of health care resources; and reducing the time lag in moving evidence into practice [4].

Evidence-based practice and the process of EBDM assume two fundamental principles:

First, scientific evidence alone is never sufficient to make a decision. Evidence-based decision making recognizes that the evidence from scientific research is only one component of the decision making process and in itself is not sufficient to inform next steps. Evidence-based decisions integrate the best scientific research evidence with evidence derived from relevant experience and judgment, patient’s preferences and values, and the clinical/patient circumstances [9]. Second, within each form of evidence, hierarchies exist and these hierarchies can guide decision making. Evidence-based decision making is a structured process that incorporates a formal set of rules for interpreting evidence. Simply put, EBP and the process of EBDM help decision makers in health care to collect and critically appraise all the best evidence for the purpose of guiding decisions. This approach is in contrast to traditional decision making, which in health care relies more on intuition and the use of information gained by consulting authorities (e.g., colleagues and textbooks) [10, 11].

#### The need for evidence-based practice in addictions

The principles of EBP are inherent in the EBDM process; this process has been described extensively by others [12–14]. The process defines a set of skills which, when comprehensively applied, produce effective decision-making. The skills needed to apply the EBDM process include: formulating focused researchable questions; finding all relevant “internal and external evidence” pertaining to the question; critical appraisal of internal and external evidence; integrating internal and external evidence; and the evaluation of the decision making process.

The development of EBP and the process of EBDM emerged from the need for a more systematic approach for improving the quality of health care in an era of limited resources [15]. Today a number of issues continue to drive the need for the continued development of competency in EBDM in the substance abuse and related addiction disorder field. These include, but are not limited to:

1. Significant delays between the time when new evidence becomes available and its application, when appropriate, to care (also referred to as the knowledge-to-action gap) [16–18].
2. Variations in practice within professions, despite significant advances in our understanding of effective prevention and treatment practices [19, 17, 20].
3. A proliferation of published studies in recent years, which has made it extremely difficult to remain current in one’s field [14].

Health care systems must be prepared for constant change if they are to provide the highest standards of care, to the largest number of people, while minimizing the ever-burgeoning costs associated with such goals [21]. Within Canada’s addiction services agencies, providing such care is dependent upon the capacity of decision-makers (e.g. senior management) to make evidence-based decisions [22, 23]. Competency in the skills defined by the EBDM process make addressing current challenges more manageable by supporting busy professionals in their capacity to find, critically appraise and integrate evidence, where appropriate. Such practice can lead to the development of effective *systems*, ones that are guided by evidence-based policies, standards, practice guidelines, programs and services [2, 4, 24]. Lack of skill in EBDM, and a failure to apply it when making decisions, may lead to the delivery of suboptimal or even ineffective programs, services, and supports, poor patient outcomes, and cost-ineffectiveness [16, 17, 25–28].

To provide a high standard of care for persons affected by substance abuse and addiction, the addictions workforce must possess knowledge of the principles of EBP and competency in the components of the EBDM process. This need is clearly defined in the competency profiles of members of all occupational clusters for Canada’s SAW [2], and the need is particularly defined for Senior Management. However, to date, evidence pertaining to the understanding of the principles of EBP or competency in the EBDM process for this occupational cluster remains anecdotal.

The objectives of the current study are: 1) To appraise knowledge of the principles of Evidence-Based Practice by Senior Management at selected addictions services agencies in Nova Scotia. 2) To investigate knowledge of the components of the EBDM process and their use in decision making by Senior Management at selected addictions services agencies in Nova Scotia.

## Methods

The methodological orientation and theory of this study was guided by the principles of directed content analysis [29], a subtype of the qualitative research approach content analysis [30]. Content analysis is an effective method for analyzing interview data, and it has been suggested as a promising, effective approach when conducting research with health-related disciplines [31]. Directed content analysis is suggested for use when exploring phenomena for which existing theory or research exists, but is incomplete and would thus benefit from further description [29]. When analyzing data using directed content analysis, a coding scheme is developed in advance, and is then applied to the data. For this study, the coding scheme was developed from the EBP literature. Additional codes were developed using an iterative process as interview transcripts were examined and analyzed. The additional codes were applied retroactively to all previously coded transcripts to ensure the coding scheme was consistent.

Interviewing is the most common method of data collection in qualitative research [32–34]. A semi-structured interview approach was determined to be the most appropriate for this study. The choice to employ a semi-structured interview was based on a review of the literature as well as a review of qualitative research studies investigating related topics such as clinical decision making, and the use of EBP. A semi-structured interview was chosen because it uses predetermined questions, but allows the participant the time and scope to express their opinions on a particular subject [35]. Further, a semi-structured interview allows the interviewer to explore complex topics of interest by adding novel questions and prompts to the existing set of interview questions as the need arises; the interviewer is not held to the exact questions as appear in the guide.

Evidence-based decision-making is a complex topic, yet it is also driven from a logical point of view. In this study, where participants had extensive experience with decision making, it was plausible to assume that they might be using a model of decision-making very much akin to that of EBP, but that the standard language of EBP would nonetheless appear foreign to them. To provide the researcher the opportunity to explore participant decision-making processes that may not have been captured in the original questions, the interviews needed to allow for deviations from the original guide. The semi-structured interview was the only option that would allow for such deviations.

Many quantitative tools for evaluating health care professional’s capacity in EBDM exist but most have yet to be validated, or are only valid for testing selected components of the EBDM process. The best researched tools; the Fresno [36] and Berlin [37] have been validated for use, however, these tools have limitations that prevented their use in the current study. The Berlin tool evaluates only one component of EBDM, skill in critical appraisal, while validated variations on the Fresno tool include the use of case scenarios whose context are not suited to the intended audience of this study [38]. Further, a recent systematic review of tools that assess EBP behavior in healthcare professionals revealed that there is only one valid tool that can assess all five components of EBDM, and it has yet to be tested for reliability [39]. The idea that a semi-structured interview technique is the most effective means of assessing EBDM competency is further backed up by a compelling argument in a recent letter to the editor in the Journal of Education for Health [40].

The Principal Investigator (PI) of the study, Matthew Murphy (MM), has experience with the qualitative interview process, and conducted the interviews for all participants. The PI held a Bachelor of Science with honours in Psychology. Analysis of the interview data was conducted by the PI and occurred simultaneously with data collection. Other members of the research team were Robert Gilbert (RG), Jayne MacCarthy (JM) and Lynda McAllister (LM): RG is an associate professor at Dalhousie University and has expertise in EBDM and applied clinical research; JM is a provincial Knowledge Exchange Facilitator and qualitative researcher with Addictions Services and LM is manager of Quality and Research Utilization with Addictions Services, Pictou County Health Authority. Members of the research team contributed equally to question development and RG contributed to data analysis.

### Participant identification and recruitment

**Figure 1:**
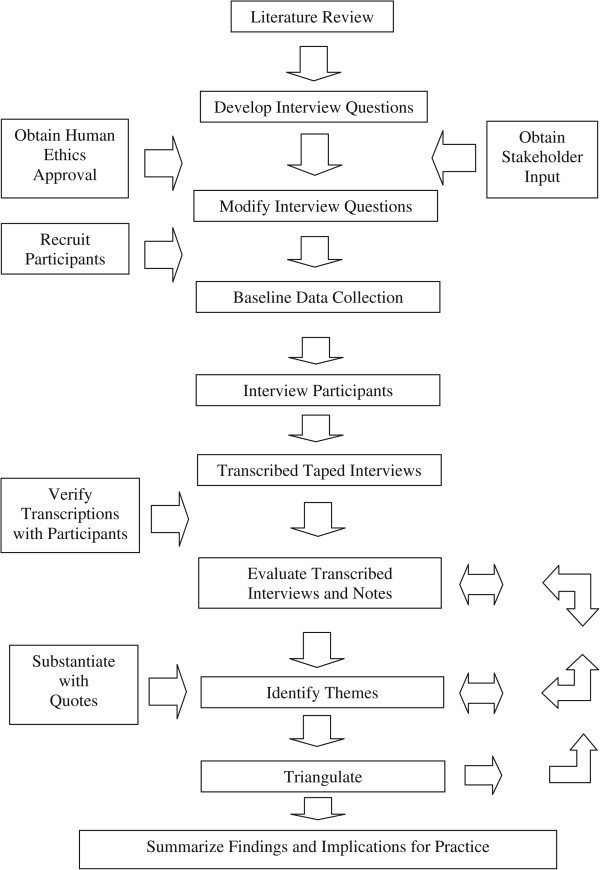
**Flow chart of research process.**

The flow diagram presented in Figure 1 illustrates the research process followed in this study. Participants in this study were recruited from selected addiction services agencies in the province of Nova Scotia using a purposeful method. The participant pool was identified by matching the role description for the occupational cluster Senior Management, with the role descriptions of people working in addiction services agencies in Nova Scotia. The descriptions and expectations for the occupational cluster Senior Management has been described in the document *Competencies for Canada’s Substance Abuse Workforce*[2] and is summarized in Table 3. In Nova Scotia, addiction services agencies jobs aligning with the above descriptions and expectations include: manager, administrative director, clinical director, and vice president. Of note, the descriptions and expectation for the occupational cluster Senior Management align with the definition for the Medical Subject Heading (MeSH) description of “Senior Administrative Personnel”.

**Table 3:** **Role description for the occupational cluster Senior Management**

Occupational cluster	Role description
**Senior Management**	Persons responsible for providing directions in all aspects of the agency’s functioning and all services it provides. Provides leadership in the development and implementation of strategic and operational plans; manages finances, HR strategy and public relations. Example job titles: Executive Director, Clinical Director, Program Director, Program Manager, Controller, Office Manager

All totaled, 47 people hold Senior Manager positions at Nova Scotia addictions services agencies [2]. Convenience sampling was used in conjunction with purposive sampling and based on previous qualitative studies [41, 42], we determined that recruiting 20 participants would exceed our data saturation requirements. All Senior Managers within a 200km radius of the PI were invited to participate. This represented all Senior Managers from 4 addiction services agencies collectively serving 57% of Nova Scotians.

To the best of our knowledge, no study has determined the educational backgrounds or professional experiences of persons working in addictions services agencies in Nova Scotia nor has there been a study to define occupational clusters within these agencies. Anecdotal evidence based on conservations with Senior Management in Nova Scotia addictions services agencies, including Gregory Purvis, Director Addictions Services, Pictou County Health Authority and co-chair of the CCSA National Advisory Group on Workforce Development, suggests that the educational background of the Nova Scotia addictions workforce is similar to that described for Canada (i.e. composed primarily of persons with professional degrees in social work, nursing, clinical psychology, counselling and medicine). Furthermore, the occupational clusters described in the document *Competencies for Canada’s Substance Abuse Workforce*[2] typify the workforce structure of addictions services agencies in Nova Scotia.

### Development of the interview guide

The interview guide (presented in Additional file 3) was developed to evaluate knowledge of the principles of EBP, and knowledge and use of the EBDM process. The development of the interview guide was based on a review of the EBP literature [14]. Initial draft questions were reviewed by stakeholders (one manager, one director, and one clinical therapist) from an addiction services agency in Nova Scotia and an EBDM expert at Dalhousie University. These individuals provided suggestions for making the specific wording of the interview questions more relevant and generalizable to the Nova Scotian SAW. Furthermore, questions contained in the interview guide were pilot tested with three members of the Nova Scotian SAW. Questions contained within the interview guide were designed to build rapport with the participant such that an opportunity would be created for gaining insight into senior management’s knowledge of the principles of evidence-based practice, and use of the process of EBDM. The one-on-one semi-structured interviews were conducted in the office of the participant, or via telephone. Interviews ranged in duration from thirty to sixty minutes. Permission to digitally record the interviews was included in the consent process. In addition to the digital recordings, a log of interview field notes was maintained for each participant interview. This log was then used during the analysis of interview data to help contextualize individual responses.

After each interview was conducted it was transcribed verbatim by MM and then returned to the participant to provide the opportunity to edit, clarify, elaborate, or revise as needed. The goal of this form of member checking is to determine if the data is congruent with the experiences of the participant, thereby increasing the validity of the data obtained [43, 44]. As with many studies that include participant interviews, permission to use individual quotations to support conclusions was obtained.

The interview data was analyzed using directed content analysis [29]. Using the themes and language of EBP, an initial list of codes was developed and applied to each transcript. As analysis proceeded, additional codes were developed and applied as necessary. Previously coded transcripts were then reviewed to see if the newly developed codes were applicable. Coding was initially conducted by hand, on a line-by-line basis, assigning a single code to each line. Where appropriate, more than one code was assigned to an individual line. After the initial hand-coding was completed, the transcripts were coded on a line-by-line basis using NVivo 9.0 Software (QSR International, California). The initial hand-coding process increased familiarity with the data and was compared (triangulated) with the results of the NVivo coding to increase the validity of the coding process [43, 44]. Once coding of all transcripts was complete, the codes were collapsed into broader categories and subsequently into even broader themes. In addition, the transcripts were reviewed by multiple members of the research team to ensure accuracy of coding and analysis (inter-coder reliability). Following transcript review, in-depth discussions were undertaken to ensure consistency in interpretation.

As suggested by Sandelowski [45] the qualitative data were reinforced by quantitative counts of the participants discussing specific themes. Table 4 lists the thematic response frequency (percentage) and the associated written descriptors.

**Table 4:** **Percentage values for terms used to describe thematic response frequency**

Term	Thematic response frequency (percentage)
Few	Discussed by less than 25%
Some	Discussed by 25 – 50%
Frequently	Discussed by 50 – 75% of participants
Majority	Discussed by greater than 75%

A variety of techniques and procedures exist that can be used to increase the validity of the data in qualitative inquiry [43, 44]. The current study employed: reflexivity; the use of an audit trail; the use of member checking; and triangulation [43].

Ethics approval was obtained from Dalhousie University Research Ethics Board (project# 2011-2447).

## Results

### Participant characteristics

Data for this study was collected between October 2011 and February 2012. A purposeful sample of 20 senior management personnel was invited to participate in this study. Eighteen persons completed the semi-structured qualitative interview. Of the two persons who did not participate, one did not appear for the initial meeting and ceased communication thereafter and a second participant agreed to the initial meeting, but subsequently decided against participating citing redundancy due to the participation of other individuals from the same agency. This sample of participants was drawn from employees of selected provincial District Health Authorities that collectively provide substance and addiction-related prevention and treatment services to approximately 57% of the Nova Scotian population [46]. The participants worked in agencies that served clients from urban and/or semi-urban and rural areas.

As described in Table 5, participants had an average (mean) of 16.5 years of experience as senior management (range of 32 years, Min = 3, Max = 35, ơ = 3.81) and had a mean age 54.3 years (standard deviation = 4.13 years). Participants held a variety of university degrees: social work, nursing, psychology, medicine, business administration, pharmacology, and community health and health promotion. Most (14) participants held a graduate degree (master’s degree or higher) and four held bachelor’s degrees. It is uncertain if this education background and number of years of experience for this sample is representative of Senior Management in addiction services agencies in the province of Nova Scotia. Four participants indicated that they had taken part in a two-day workshop on EBDM, and 11 participants had received training through their library services department to aid in basic literature search strategies (i.e., how to use electronic databases, how to use MeSH, etc.,).

**Table 5:** **Demographics of participants in this study**

Senior management (n = 18*)	
**Gender**	
Female	n = 8
Male	n = 10
**Highest Level of Education Obtained**	
Bachelors	n = 4
Masters or higher	n = 14
Background Education**	
Nursing	n = 2
Social Work	n = 6
Clinical Psychology	n = 5
Business Admin.	n = 2
Pharmacology	n = 1
Community Health	n = 1
Medicine	n = 1
Health Administration	n = 1
Nurse Practitioner	n = 1
Mean years in Senior Management	; = 3.81
	range = 3–35 years

*Although one individual’s job title within the DHA was that of clinical supervisor, their role within the DHA matched that of the role description for the Occupational Cluster Senior Management.

**One participant held both a masters of nursing as well as a nurse practitioner diploma; one participant held masters degrees in both business administration and pharmacology; this information is included twice.

### Interview results

Semi-structured qualitative interviews were conducted with 18 senior managers from addiction services agencies in Nova Scotia. Data saturation in this study occurred during the thirteenth and fourteenth participant interviews. To guarantee that saturation had occurred, the remaining four participant interviews were conducted. These interviews helped to substantiate previously identified themes, but did not contribute to the identification of new themes.

**Figure 2:**
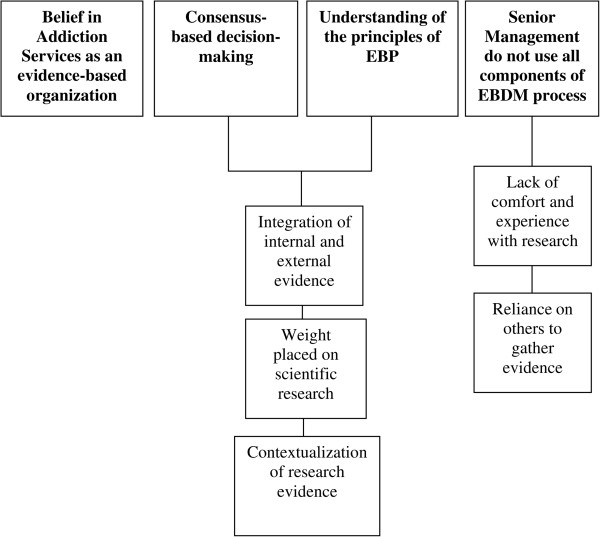
**Flowchart of themes based on directed-content analysis.**

Analysis of the participant’s interviews revealed four major themes: 1) Senior Management believe that their agencies develop and provide programs, standards, policies and services that are evidence-based; 2) Decisions pertaining to the development of addiction services agencies programs, standards, policies and services (*systems* components) are made through consensus; 3) Senior Management understand the principles of EBP; and 4) Senior management themselves do not use all component of the EBDM process when making decisions, often times delegating components of this process to decision support staff. Figure 2 presents the four major themes, as well as their associated minor themes.

***Theme 1: There is a belief among Senior Management that addiction service agencies in Nova Scotia develop and provide evidence-based programs, policies, standards and services.***

The majority of Senior Management described their agency as evidence-based. As one participant stated (S16):

[…] we’re running an evidence-driven, evidence-informed program, we pride ourselves on research, it is one of our big selling points, we use evidence where others don’t, I’m big on “show me how you use it, and prove that you maintain it”.

Another participant referenced the use of evidence in their ability to defend decisions or actions to the public (S6):

… because we have done such good work on the research and evidence we are able to explain to people in the community why we are doing things, why something is important, what the issues are, and can back it up with the research and the evidence.

In contrast, a few participants believed addiction services agencies are not evidence-based. According to one participant (S8) “everyone talks a really good game around evidence-informed, evidence-based, evidence-this evidence-that, but in practicality I don’t think we have really hit a place where we really do something meaningful with it”.

***Theme 2: Decisions pertaining to the development of addiction services agencies’ programs, standards, policies and services are made by using a consensus process e.g., they gather together their experiences and knowledge and then come to decision through consensus.***

Decision-making, as described by the majority of Senior Management, was reached through consensus, although not every decision is arrived at through that process. Participants frequently made reference to bringing needs to one ‘table’ or another, where decisions are made through consensus. According to one participant “[w]ell I sit on a number of decision-making bodies around [treatment X] and we do have processes and our goal is consensus” (S14).

Similarly, another participant stated (S3):

[t]here is an effort to come to some sort of consensus regarding the decision-making because it is a group process. So there is discussion trying to move folks to that direction, if consensus isn’t achieved then the decision will be put to a vote…

Arriving at decisions through consensus is compatible with the principles of EBP provided that all components of the EBDM process are inherent to the decision-making, applied comprehensively and through the use of defined skills.

***Theme 3: Senior Management understands the principles of EBP.***

Evidence-based practice is based on two principles: 1) The recognition that scientific evidence alone is insufficient to guide decision making; and 2) Within available sources of evidence, hierarchies exists. The majority of participants demonstrated an understanding of both principles of EBP. The recognition that scientific evidence alone is insufficient to guide a decision is exemplified by one participant who stated (S6):

[…] so research based randomized clinical trials, meta-analyses, Cochrane Reviews, and we try to temper that with the other fields of knowledge, so what are the folks who are actually doing the work saying? What’s the [client] saying?

Recognition of the hierarchy of evidence is present in the following excerpt from one participant discussing the need to gather high quality evidence (S10):

[our manager of research] has done a lot to actually help us learn that when you say ‘evidence’ it’s not a Google search, it’s something from the Cochrane Database or it’s CINAHL, or somewhere a little more reliable when we are actually searching for the evidence.

***Theme 4: Senior Management themselves do not use all components of the EBDM process when making decisions, often times delegating different components of this process to decision support staff.***

While knowledge of the principles of EBP is essential, judicious decision making also requires the comprehensive use of the EBDM process.

### Development of focused researchable question

Participants were asked to describe the process they use to define a problem, or ensure that when developing a question it contains all of the required components needed to inform a subsequent systematic literature search. The majority of participants stated that, at least initially, they were unsure if the question being posed was in fact the right one and that over time the question either changed or became more refined. According to one participant “in a lot of cases you just don’t know if you are asking the right question. For myself, generally something comes to mind that I am wondering about and then I start exploring it” (S7). Another participant explained that “I think the questions evolve, I don’t think you start with a perfect question, but that they evolve as you get more and more information available to you” (S2). Of the 18 participants, one described using a formal process (i.e. Population, Intervention, Comparison, Outcome “PICO”) for defining a researchable question, although this participant did not describe all of the components of that particular process.

Some participants discussed the need to consider an outcome when developing a question. For example one participant stated (S2) “I’m thinking of acupuncture, and I don’t know if I am going to get at this or not but the question for acupuncture is “is it effective, does it have good outcomes?” Another stated “obviously you are thinking of an outcome, so I would think ‘this is what I want for my outcome, what do I need to ask, and do, and go through to come up with my question to reach this point?’”(S8).

### Search strategies for gathering evidence

Participants were asked to describe strategies they used to gather evidence, including the sources they might access. Some participants discussed using bibliographic databases (i.e., PubMed and Cochrane) as a source of scientific research literature. Participants frequently indicated they did not use systematic reviews. The majority of participants identified expert opinion as a source of evidence. The majority also indicated that existing policies from other jurisdictions were considered an excellent source. The participants were not asked if they used a process to determine if other jurisdiction’s policies were created using an evidence-based approach. The majority of participants did not identify their own experience as a source of evidence; a minority did not identify the preferences and values of their patient’s/population’s as sources of evidence.

In terms of gathering evidence to answer questions or address issues, the majority of participants stated that they did not gather evidence themselves; rather they had staff members perform this function. The majority of participants cited time as one of the reasons that they did not perform their own searches; two participants also added that they were not comfortable with their own skills to perform such a search. One participant described the difficulty encountered with trying to track down the full text version of an article, rather than simply using the abstract (S10):

I find it difficult because I have never gone beyond, that’s my personal experience [..] I emailed the librarian to ask if she could help me get [this article] because I couldn’t seem to [access] it, and she sent me something back and still I couldn’t get it.

Some participants stated that they had people in positions (e.g. Research and Statistical Officers, decision support staff) to do such work and they trusted that they were experts in that area. According to one participant (S12):

[…] we have our decision support person. So we put people in place whose job is to have that expertise, so we would ask them to give us the advice, or well the literature, the evidence, and then we determine from the evidence what our approach [will be].

### Critical appraisal of external and internal evidence

When participants were asked how they would evaluate the validity of information gathered as part of their decision-making process, responses varied. Some participants stated that they would look to see if the information came from a recognized source, such as the Centre for Addiction and Mental Health (CAMH) or a well-known journal. Some participants stated that they were not comfortable performing such an evaluation and would rely on the expertise of someone else from within their agency, such as a Research and Statistical Officer, or someone in a decision support role. Some participants stated that the first part they would consider when appraising information would be the methodology. No participant described how he or she would perform such a process (participants were not prompted to do so). When prompted, participants did not acknowledge use of assessment guidelines (i.e. CONSORT, PRISMA, COREQ, etc).

A few participants discussed the need to evaluate the specific statistical tests that were employed in a study, though one commented that they did not have the requisite skills to do so “I have taken some statistics courses before but I am certainly not capable of comparing specific statistical analyses against one-another” (S9).

### Integration of internal and external evidence

Participants were asked to describe how they applied the results of their evidence gathering to their decision making process. The majority of participants described the consideration of both internal and external evidence when making a decision. According to one participant prior to deciding to implement a new program (S4):

[…] I would want to read the research on it, have a look at the outcomes, and I’d want to compare where and who it was implemented with to see if it is compatible with our area and our resources. I’d probably talk to someone who has done the program in another place, make a phone call, talk to staff about it get their views because they are great at finding stuff that I wouldn’t even think about[…].

When discussing the integration of internal and external evidence participants frequently emphasized the importance placed on scientific research to support decisions.

A few participants identified the need to gather input from relevant high quality literature, expert opinion, clients, and staff, and one individual identified the need to consider previous experiences (S17):

So when I can say ‘OK, this is what the literature says about this; this is what the experts say about this; this is what I read in this or that book; this is what our past experiences with this have been.

The majority of participants also discussed the need to adapt evidence so that it could be applied to their local context. Financial and resource limitations were cited by the majority as significant factors for consideration. According to one participant (S10):

[…]sometimes it is difficult to translate the best practice to create on the operational side of things simply because you may not always have the resources or capabilities to fully meet the standard that the best practice is setting.

### Evaluating outcomes of applied decisions

Once evidence has been integrated, contextualized and applied to a decision, an evaluation of that decision must be made to determine the outcome as well as areas for future improvement.

When asked what methods participants would use to assess the outcome of applied decisions, the majority of the participants stated that they should measure treatment and prevention outcomes, but evaluation is most often based on client satisfaction. Some of the participants acknowledged that these outcomes should be determined in advance to avoid a biased evaluation, but many participants stated that this process does not currently take place.

## Discussion

The competency profiles ascribed to Canada’s SAW demonstrate a need for understanding the principles of EBP and for possessing skill in all components of the EBDM process [47]. While all contributors to the addictions prevention and treatment *system* must be versed in these attributes, competency is particularly important in those working in the Senior Management occupational cluster. Ultimately, it is these individuals who are responsible for decisions, and the hiring of individuals who contribute to decisions, integral to the development and implementation of judicious and evidence-based policies, programs, standards and guidelines that support the treatment and prevention of addiction. The objective of this study was to appraise understanding of the principles of EBP, and investigate the knowledge and use of the components of the EBDM process during their decision making. Results suggest that Senior Management understand the principles of EBP. However, most do not themselves enact all components of the EBDM process during decision making and more than half disclosed a lack of comfort and experience with evaluating existing research. When prompted to describe their decision making process, components of the EBDM process (question formulation, search strategies, critical appraisal or outcome evaluation) were not consistently described. An apparent strength of this occupational cluster is their knowledge and described experience with integration of various forms of evidence.

The finding that most participants did not describe all components of the EBDM process might be attributed to the fact that some delegate components of the decision making process (e.g. systematic literature searching and critical appraisal) to other staff members. In fact, many participants described decision making as a team process. While this may explain, in part, the absence of description of these components, it highlights an area in need of attention. Individuals identified as providing decision support (RSOs, knowledge exchange facilitators, librarians, and others) are not an occupational cluster of Canada’s SAW and, as such, have no consistently defined roles or expectations. In the absence of competency profiles, there can be no assurance that those providing decision support understand the principles of EBP or use the EBDM process, and therefore it is unknown whether information being provided to Senior Management represents all best available internal and external evidence. Development of the occupational clusters for Canada’s SAW [2] to include decision support workers is warranted. Clearly defined competency profiles, role descriptions, and examples of indicators of competency should be developed for the occupational cluster “Decision Support Worker”. This profile would, of necessity, include the expectation of a high level of competency in EBDM.

The roles and responsibilities of Senior Management are substantial and the need to hire others to search for and appraise evidence may be an acceptable reality. During the hiring process, it would be important to judiciously assess for the ability of a candidate to understand and apply the EBDM process. Such judicious assessment necessitates the interviewer to have competency in the field. In this study some Senior Managers described difficulty in performing selected components of the EBDM process. Therefore, it could be argued that Senior Management may not have the ability to hire or recruit decision support persons with the necessary skills and knowledge.

A second area potentially in need of attention is in evaluating outcomes of applied decisions. This is a vital step to EBDM enabling the decision maker to assess decisions for effectiveness and/or identification of areas in further need of improvement. It would appear that the most common approach to evaluation in addiction services agencies has been to measure client satisfaction with service delivery and not to evaluate the specific outcomes of programs, services and supports. One possible explanation for this practice is that comprehensive outcomes are often not clearly identified prior to and during the development of *systems of care* components. Another contributor to the lack of outcome monitoring may be a lack of resources (e.g. finances, personnel) available within the addictions prevention and treatment *system* to support this type of evaluation. Regardless of the reason, in the absence of comprehensive outcome evaluation, *systems* cannot be considered evidence-based. Furthermore, in the absence of outcome evaluation, applied decisions might result in the provision of ineffective or even harmful practices.

Other themes that emerged during the study were the belief that programs, services, policies and standards offered by addictions services agencies are evidence-based and also that decisions are made through consensus. Application of the principles of EBP requires defined skill sets and includes five components: development of a researchable question, development and implementation of an appropriate search strategy, critical appraisal of internal and external evidence, integration of various evidentiary sources, and outcome evaluation. For a decision to be truly evidence-based all of these components must be integrated. The fact that most participants did not describe using all components of the EBDM process, or described challenges with certain components (i.e. including critical appraisal, and evaluation of outcomes post decisions) draws into question some participants' ability to judge addiction services agencies as being evidence-based.

This study profiles a sample of persons working as Senior Managers in selected jurisdictions of Nova Scotia. Qualitative methodology does not allow generalizabilty and therefore these findings cannot be extrapolated to Senior Management in other jurisdictions within Nova Scotia or Canada. It is of note however that, while no study has examined the educational backgrounds or professional experiences of persons working in addictions services agencies in Nova Scotia, anecdotal evidence suggests that the educational background of the Nova Scotia addictions workforce is similar to that described for Canada [48]. Furthermore, the occupational clusters described in the study, *Competencies for Canada’s Substance Abuse Workforce,* typify the workforce structure of addictions services agencies in Nova Scotia.

A recent Canadian study [26] which used similar methodology explored the use of EBDM among Canadian addiction service professionals working in agencies serving women. The study looked at the types and sources of evidence that decision makers report using; how decision makers at different levels within an organization report using research evidence; and factors that influence the use of EBDM. The researchers found that decision makers reported using: research evidence; best practice guidelines and perceived best practices; local program evaluations; client needs assessments; expert opinion; personal professional experience; and personal experiences of addiction and recovery [26]. In the current study, participants also cited use of a number of possible evidentiary sources. However, participants frequently cited placing emphasis on research evidence from published scientific literature. Participants in the study by Jack and colleagues [26] did not display a preference for any one source of knowledge, and “no one type of knowledge was considered to have greater relevance or impact” [p.6] on the decision to adopt a particular practice or treatment option. One possible explanation for the difference in preference of evidentiary sources could be the difference in participant populations. In the study by Jack and colleagues [26] participants were employed primarily in community-based organizations whereas in the current study participants came from provincial District Health Authorities. Community-based organizations tend to offer services on a not-for-profit basis, operating outside the scope of government organizations and receive funding through myriad sources. District Health Authorities receive funding from, and are responsible to, the provincial Department of Health and Wellness. Previous research has suggested that individuals working for community-based organizations show a preference for data collected locally because it is perceived as more relevant to the local context than are published research findings [49, 50]. It is also possible that the findings from Jack and colleagues [26] represent a lack of participant knowledge and skill relating to the principles of EBP. However, because Jack and colleagues [26] did not measure or evaluate their participant’s knowledge of EBP principles, or the knowledge and use of the components of the EBDM process during decision making, such comments are speculative. Likewise, Jack and colleagues [26] investigated the reported barriers to the use of evidence in decision-making, with participants reporting a lack of time, competing priorities within the workplace and a significant gap between research and practice. However, it may have been informative to have known the capacity of the decision-makers to apply the principles of EBP prior to knowing the self-identified barriers in using evidence.

No other studies have evaluated the knowledge of the principles of EBP or knowledge and use of the components of the EBDM process in Canada’s SAW. To date, studies in the addiction-related field have primarily investigated the implementation and use of evidence-based practices. These studies report a consistent gap, as much as 15 years, between what current research indicates as best practice, and what is practiced in the field [25, 51–53]. A number of the studies in this area have also specifically examined knowledge transfer and exchange strategies with the goal of identifying specific strategies to increase the uptake of evidence-based practices [54, 55]. Dobbins and colleagues [56] developed a framework for the dissemination and utilization of research for healthcare policy and practice. They identified numerous variables related to this concept including innovation, organization, environment and the individual. With regard to the individual, they identified a number of variables that could act as barriers to the use of research evidence including: a perception that research findings are not relevant to one’s context; perceived availability of research evidence; and limited critical appraisal skills.

The findings from the current study corroborate only one of these variables: participants did discuss a lack of comfort with critical appraisal skills. However, in contest to the other two variables suggested by Dobbins [56], participants in the current study clearly articulated awareness of research and demonstrated the ability to contextualize findings to their locale.

A variety of techniques and procedures exist that can be used to increase the validity of the data in qualitative inquiry [43, 44]. The current study employed: reflexivity, the use of the constant comparison method, the use of an audit trail, the use of member checking [43], and the use of triangulation (inter-coder reliability, independent verification of themes). As described above, the results of qualitative research are not intended to be generalizable, rather they provide a snapshot of a given jurisdiction or group of individuals. A potential limitation to this study is in the sampling method. Sampling was based on convenience (proximity to the PI) and therefore may have led to some form of selection bias. However, the results of this study show that the methods employed are a feasible means of capturing such data, and may act as a pilot for larger provincial or national study.

## Conclusions

This study has identified the need to consider identification of an additional occupational cluster for Canada’s SAW, one which defines the expectations and competencies of persons specifically hired to provide decision support. It also provides insight into the challenges some Senior Managers face when performing selected components of the EBDM process. These findings were not unexpected. The majority of study participants completed formal education many years prior to the integration of EBP and a formalized EBDM process into curricula of university programs. Furthermore, effective programs for professional development pertaining to EBDM are limited. This study advocates for the provision of professional development training opportunities to support Senior Managers in their EBDM and when hiring decision support staff. Such initiatives will support the continued development of evidence-based *systems*.

## Electronic supplementary material

Additional file 1: **Senior management: behavioural competency indicators linked to evidence-based practice.** (DOCX 17 KB)

Additional file 2: **Interview guide.** (DOCX 15 KB)

Additional file 3: **CCSA Copyright Permission Letter.** (DOCX 332 KB)

## Abbreviations

CAMH: Centre for addiction and mental health
CCSA: Canadian centre on substance abuse
EBDM: Evidence-based decision-making
EBM: Evidence-based medicine
EBP: Evidence-based practice
PI: Principal investigator
SAW: Substance abuse workforce.
