# Supplementary material for: Application of the principles of evidence-based practice in decision making among senior management in Nova Scotia’s addiction services agencies
Source: Subst Abuse Treat Prev Policy. 2014 Dec 5;9:47. doi: 10.1186/1747-597X-9-47 (PMC4320476; doi:10.1186/1747-597X-9-47)
Supplement: Supplementary file 1 — Additional file 1: Senior management: behavioural competency indicators linked to evidence-based practice. (DOCX 17 KB) [file 13011_2014_323_MOESM1_ESM.docx]

APPENDIX A Senior Management: Behavioural Competency Indicators Linked to Evidence-Based Practice

| **Competency: Adaptability/ Flexibility** |
| --- |
| - Gathers information from a variety of sources to assess changing plans and priorities and makes informed choices based on available information - Prepares for change and adapts own plans and priorities accordingly - Provides advice and guidance to others to assist them in adapting to difficult or changing situations |

| **Competency: Analytical Thinking and Decision Making** |
| --- |
| - Consults others, researches information and determines   relevant patterns or trends to understand the issue or problem and identify potential causes   - Identifies multiple courses of action, considering who may be affected by a decision as well as potential outcomes - Evaluates the advantages, disadvantages and effectiveness of alternate approaches and possible courses of action - Identifies potential problems or risks associated with a decision or action and uses critical thinking to implement plans that mitigate their effects - Uses logic, past relevant experience, lessons learned and evidence-based criteria when forming conclusions and making decisions - Determines when to act quickly/decisively and when to deliberate on or contemplate decisions - Makes informed and timely decisions to determine a course of action in complex, ambiguous or urgent situations - Makes decisions in alignment with organizational values and directions - Develops creative, forward thinking options and recommendations, soliciting opinions of others to gain different perspectives - Makes decisions based on evidence-based practice, reasoning and clinical experience and in consultation with key stakeholders as appropriate - Identifies and respectfully challenges judgment or decision making that is unclear or unsupported - Evaluates the effectiveness and efficiency of a solution after implementation - Identifies the potential impacts that trends or events may have on services, clients and/or employees |

| **Competency: Continuous Learning** |
| --- |
| - Takes responsibility for one’s own learning and professional development - Self-assesses and seeks feedback from others to identify skills and knowledge gaps and seeks to close these through self-study, continuing education and seeking assistance or advice, and coaching - Keeps up to date with current research, literature and other developments relevant to the field and applies learning to one’s practice - Draws on the knowledge of others through networking, teamwork and partnering - Actively pursues information, competency-based and other learning opportunities, beyond current job role or area of expertise, that add value in current position - Seeks learning opportunities in rapidly evolving and emerging subject areas within and peripheral to one’s professional practice - Participates in research to advance the knowledge in the field - Actively contributes to building a learning culture, encouraging learning and knowledge sharing and advocating for professional development activities - Supports and/or supervises others in their learning and professional development by providing feedback, coaching, mentoring and resources, and by identifying learning goals and opportunities for professional development |

| **Competency: Creativity and Innovation** |
| --- |
| - Implements alternate evidence-based techniques and approaches rather than using the same solution repeatedly for all clients - Based on evidence-based practice and drawing upon a broad empirical and theoretical knowledge base, adapts existing approaches and techniques to meet unique, situation-specific needs - Creates new ideas, solutions or approaches to ongoing challenges and problems - Explores best current knowledge in the field and adapts and applies this knowledge to reflective practice as a source of inspiration and insight into new options and solutions - Draws correlations between seemingly unrelated issues and ideas and identifies what is not apparent to others - Develops innovative, contextually relevant intervention methodologies that incorporate both the rigour of research and the shared experience of practitioners and clients - Effectively facilitates brainstorming activities |

| **Competency: Developing Others** |
| --- |
| - Evaluates group learning needs and plans group developmental activities based on sound evidence and experience |

| **Competency: Effective Communication** |
| --- |
| - Integrates and synthesizes information from appropriate sources into written work - Practices knowledge exchange principles in both written and verbal communication (simple, clear, direct, respectful, timely, evidence-based) - Synthesizes complex documents and ideas from multiple sources into written material |

| **Competency: Self Care** |
| --- |
| - Develops self care best practices that will assist self and peers in coping with work challenges such as stress, fatigue and difficult situations e.g., the appropriate use of humour to relieve tension |

| **Competency: Ethical Conduct and Professionalism** |
| --- |
| - Incorporates best practice knowledge into work whenever possible |

| **Competency: Leadership** |
| --- |
| - Conducts needs analyses to determine if change is necessary, and identifies and implements change strategies |
