# Supplementary material for: Application of the principles of evidence-based practice in decision making among senior management in Nova Scotia’s addiction services agencies
Source: Subst Abuse Treat Prev Policy. 2014 Dec 5;9:47. doi: 10.1186/1747-597X-9-47 (PMC4320476; doi:10.1186/1747-597X-9-47)
Supplement: Supplementary file 2 — Additional file 2: Interview guide. (DOCX 15 KB) [file 13011_2014_323_MOESM2_ESM.docx]

APPENDIX B Interview Guide

1) What kinds of information do you use that you would consider as evidence when making decisions?

2) Do you use a structured process or model when making decisions in your work?

3) How do you make sure you are asking the right question?

4) When collecting information/evidence how do you know when to stop? When you have it all?

Prompt: A few minutes ago we discussed a list of evidence sources, you identified several that you use in your decision-making processes. I would like to discuss the ease of access concerning these sources.

5) How do you know the information provided in these publications (studies, reviews) is strong?

*Prompt: Let’s create a scenario, a new program guideline is going to be introduced in your workplace and you have been asked to evaluate it before it goes live, how would you do this?*

6) How do you determine that you are answering the question you set out to answer?

*Prompt: How do you evaluate the outcome to make sure you are doing what you set out to do?*

7) What are some of the barriers you might face when using evidence in the decision-making processes of your organization?

8) Are there any specific facilitators to using evidence in decision-making processes in your organization?

9) Do you have any further comments concerning what was discussed? Anything else you would like to add?
