# Supplementary material for: Application of the principles of evidence-based practice in decision making among senior management in Nova Scotia’s addiction services agencies
Source: Subst Abuse Treat Prev Policy. 2014 Dec 5;9:47. doi: 10.1186/1747-597X-9-47 (PMC4320476; doi:10.1186/1747-597X-9-47)
Supplement: Supplementary file 3 — Additional file 3: CCSA Copyright Permission Letter. (DOCX 332 KB) [file 13011_2014_323_MOESM3_ESM.docx]

APPENDIX C CCSA Copyright Permission Letter
